# Predicting Difficult Airway Intubation Based on Maxillofacial Trauma: A Retrospective Study

**DOI:** 10.7759/cureus.24844

**Published:** 2022-05-09

**Authors:** James Yang, Aakash Trivedi, Zoraida Alvarez, Ratul Bhattacharyya, Felippe Sartorato, Francesco Gargano, Benjamin Rebein, Jamshed Zuberi

**Affiliations:** 1 General Surgery, St. Joseph's University Medical Center, Paterson, USA; 2 Surgery, St. Joseph's University Medical Center, Paterson, USA; 3 Basic Biomedical Science, Touro College of Osteopathic Medicine, Middletown, USA; 4 Trauma and Acute Care Surgery, St. Joseph's University Medical Center, Paterson, USA

**Keywords:** retrospective study, facial fracture, trauma, difficult intubation, maxillofacial trauma

## Abstract

Purpose

The purpose of this study was to determine which types of facial injuries in traumatic patients' wounds cause difficult intubation for anesthesiology team. By anticipating potential complications with airway management, the surgeons can be better prepared for emergent cricothyrotomy if needed. This could include prior to the planned procedure in the operating room (OR) as well as in emergent conditions in trauma bay.

Methods

Trauma patients with facial injuries in a level II trauma center from January 2007 to September 2017 that required intubation were evaluated for types of facial injury. Anesthesiology intubation documents were reviewed to determine which types of facial injuries were associated with difficult intubation per anesthesiology documentation.

Results

A total of 232 subjects were selected and it was found that patients with LeFort II facial fracture, bilateral mandibular fracture, and facial fracture associated with basilar skull fracture were noted to have difficult intubation by the anesthesiology team.

Conclusion

On the basis of CT imaging findings, our study demonstrates that certain types of facial fractures could pose difficult intubation. Surgeons should be aware of these injuries and be ready to intervene with emergent cricothyrotomy if necessary.

## Introduction

Airway management is considered the first step in the management of trauma patients. Failure to secure or protect the airway is the most common cause of patient mortality in trauma patients with facial injuries [1-3]. We aimed to categorize which types of facial traumatic injury will cause difficult intubation. A retrospective study was performed at a level II trauma center using subjects with a traumatic facial injury (aged 18 years and above) from January 2007 to September 2017 that required intubation. This retrospective study could provide insight into anticipated difficulties in intubation correlating to specific facial injuries.

## Materials and methods

A retrospective study was performed on all subjects ages 18 and above from January 2007 to September 2017, who presented to the trauma bay with facial trauma that required a maxillofacial computerized tomography (CT) scan and intubation at a high-volume level II trauma center. The charts of all subjects were reviewed via anesthesiology documentation and the intubations were classified as difficult or non-difficult. Difficult intubation per the American Society of Anesthesiologists is defined as intubation that requires three or more attempts when using an average laryngoscope or when endotracheal intubation takes longer than 10 minutes. Intubation included both emergent intubation in the trauma bay as well as elective intubation prior to a scheduled operation. Based on available data, we were unable to distinguish the number of emergent intubation vs elective intubation. The CT scans of the subjects that were classified as difficult intubation were reviewed and categorized into specific types of injuries. These injuries included LeFort II facial fractures, bilateral mandibular fractures, facial fractures with associated basilar skull fracture, and facial fractures with associated complications. These complications included airway edema, blood in the airway, and lack or loss of teeth.

## Results

A total of 232 subjects, presented as trauma patients with traumatic facial injuries from January 2007 to September 2017, were selected for the study from a level II trauma center in an urban city. Each of the selected patients has had undergone intubation during the hospital stay as well as a head or maxillofacial CT imaging. Of these, a total of 56 subjects (24%) were classified as patients with difficult intubation per anesthesiology documentation. CT imaging revealed 32 subjects out of the total number of difficult intubations (57%) with LeFort II facial fractures, 12 (21%) with bilateral mandibular fractures, eight (14%) with facial fractures and associated basilar skull fractures, and four (7%) with facial fractures and associated complications such as airway edema, blood in airway, and lack or loss of teeth (Table 1). 

**Table 1:** Types of maxillofacial traumatic injury that caused difficult intubation

Types of maxillofacial trauma	Number of difficult intubations	Percentage of difficult intubation (%)
LeFort II facial fx	32	57
Bilateral mandibular fx	12	21
Facial fx with associated basilar skull fx	8	14
Facial fx with associated complications such as airway edema, blood in airway, lack/loss of teeth	4	7

## Discussion

Facial injuries are commonly seen in the population with traumatic injuries. Further, 15-25% of trauma patients are estimated to have facial trauma [4]. These injuries can range from lacerations and bruises to fractures of facial bone. The more severe injuries could cause difficulties with airway management which is critical in management. Presented retrospective study of 232 subjects who presented with traumatic facial injuries demonstrated that LeFort II facial fractures, bilateral mandibular fractures, facial fractures with basilar skull fracture, and facial fractures with associated airway edema, blood in airway, and lack or loss of teeth had caused difficult intubation by the anesthesia team.

**Figure 1:**
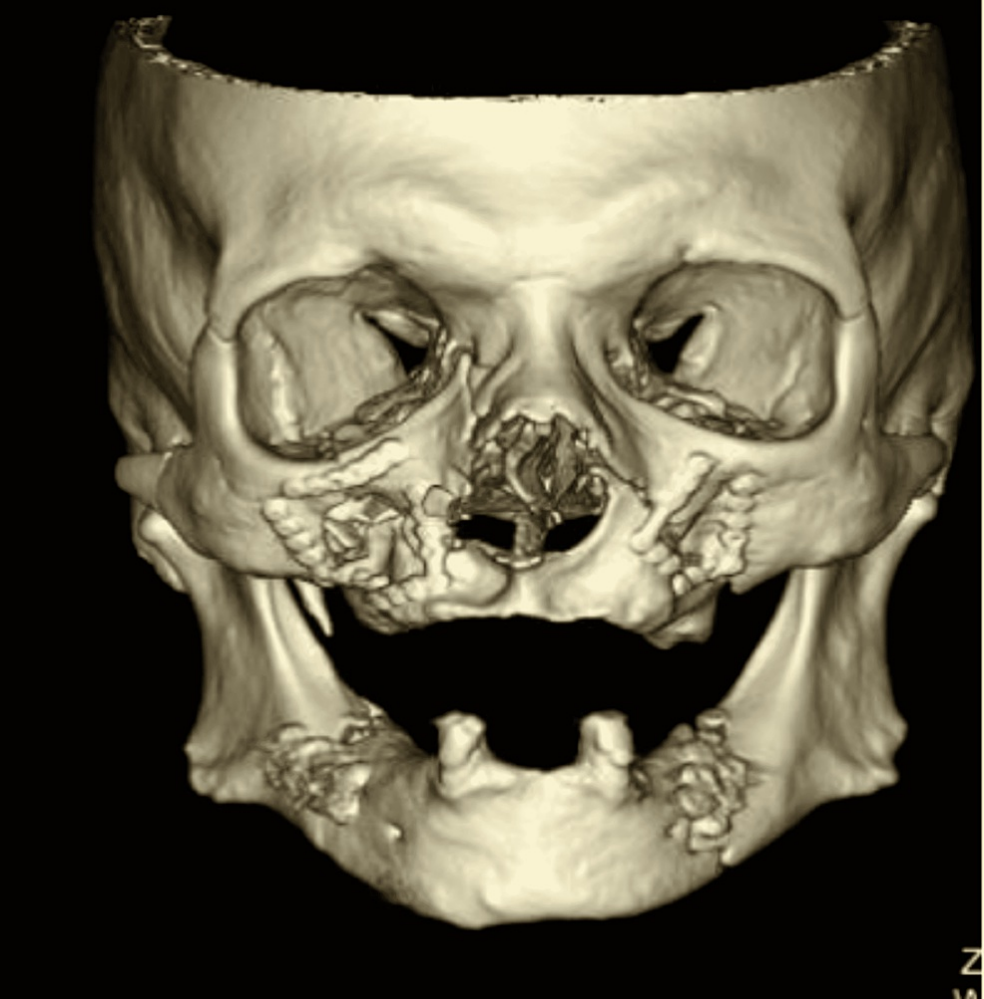
Three-dimensional (3D) CT post-open reduction internal fixation of LeFort II and bilateral mandibular fracture

Of the facial injuries that caused difficult intubation, LeFort II facial fractures were most commonly seen (32 out of 56 difficult intubation). LeFort classification system categorizes facial fractures based on its plane of injury. A LeFort I fracture a transverse fracture going through the maxillary sinuses, lower nasal septum, and pterygoid plates often caused by direct horizontal impact to the upper jaw. A LeFort II fracture is an oblique fracture that crosses the zygomaticomaxillary suture, inferior orbital rim, and nasal bridge often caused by direct impact to the central midface (Figure 1). Lastly, a LeFort III fracture is a fracture above the zygomatic arch and through the lateral and medial orbital walls as well as nasofrontal suture and is associated with craniofacial dissociation [5,6]. LeFort II fractures can cause posteroinferior displacement of the maxilla towards the base of the skull and block nasopharyngeal airway [1].

**Figure 2:**
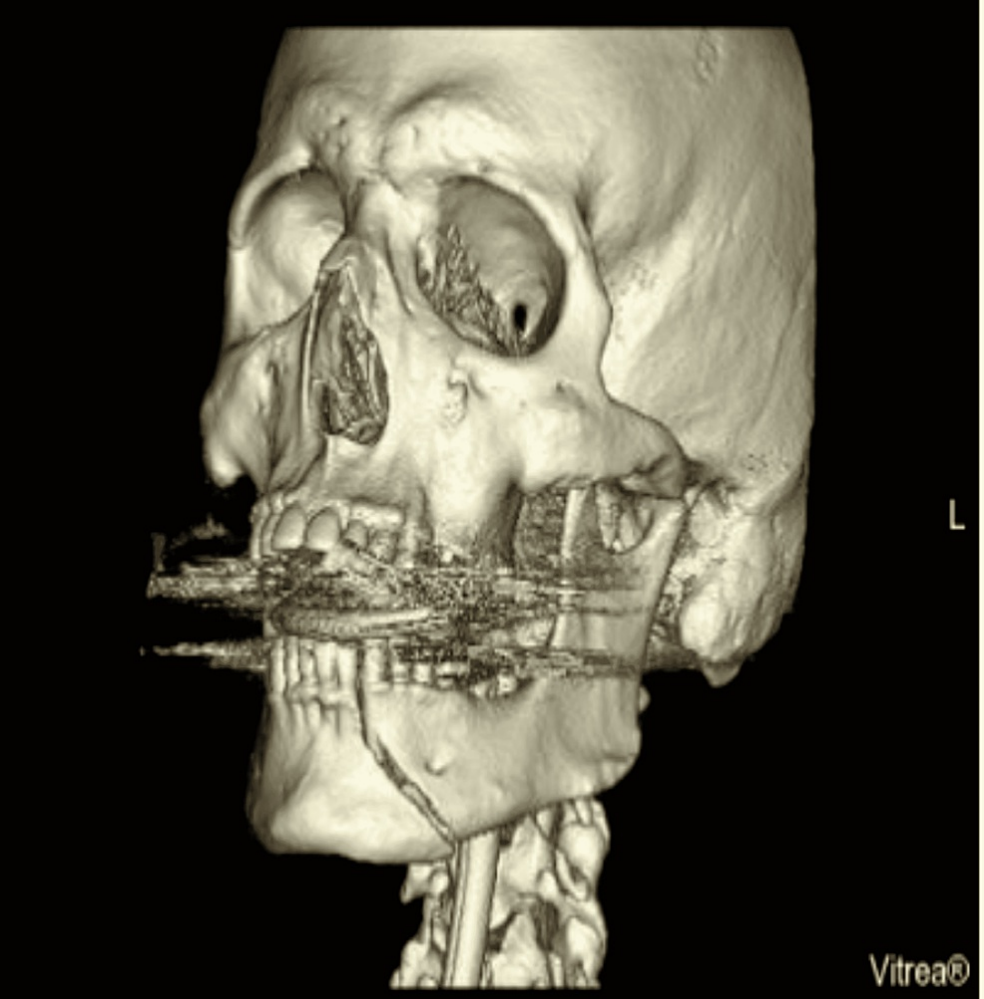
Three-dimensional (3D) CT left lateral view of bilateral mandibular fracture

**Figure 3:**
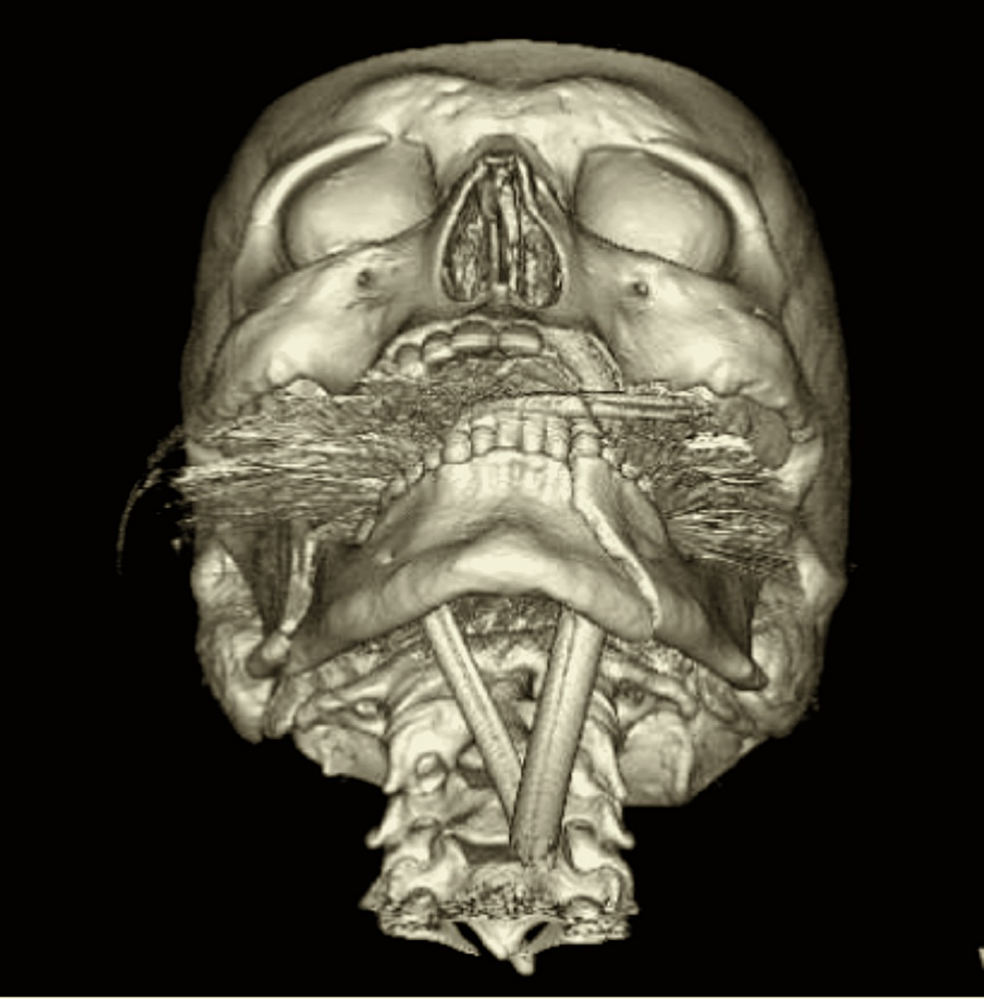
Three-dimensional (3D) CT anterior view of bilateral mandibular fracture

Bilateral mandible fracture and facial fractures associated with basilar skull fracture were the second and third most common causes of difficult intubation, respectively (Figure 2). Bilateral mandible fracture can cause posterior tongue deviation and block the oropharynx causing difficulties in the placement of endotracheal tube (Figure 3) [1]. Basilar skull fractures can be a relative contraindication to nasopharyngeal airway. These patients often have other associated facial fractures that require surgical fixation. Nasal intubation is preferred over oral intubation during these operations so fiberoptic-assisted nasotracheal intubation may be required [7].

Approximately 0.1-10.1% of intubations are classified as difficult intubation [8]. Our study of 232 trauma patients with facial injuries demonstrated that 24% of the patients had intubation classified as difficult. Surgeons, especially trauma surgeons, must be aware of injuries that could potentially pose difficult intubation by anesthesiology colleagues. Laryngeal mask airways can be used as temporizing measure but surgeons must always be prepared for cricothyroidotomy to ensure proper airway.

## Conclusions

Current practice essentials in maxillofacial trauma include airway management via artificial airways and/or surgical fixation. Types of facial injuries as well as associated complications to the injuries create difficult intubations in many of these patients. By reviewing the CT scans, we were able to identify which types of facial injuries could pose difficulty in intubation. These injuries included LeFort II facial fractures, bilateral mandibular fractures, facial fractures with associated basilar skull fracture, and facial fractures with associated complications such as bleeding and edema. This retrospective study provides insight into anticipated difficulties in airway management (emergent or elective intubation for surgical procedure) correlating to specific facial injuries and their associated injuries. Further study can be done to demonstrate which percentage of specific facial fractures, such as LeFort II fractures, are associated with difficult intubation. Surgeons should always be prepared for emergent cricothyrotomy if necessary.
